# Draft genome sequence of *Lactococcus lactis* IBB2, a candidate probiotic strain isolated from bovine milk

**DOI:** 10.1128/mra.00846-25

**Published:** 2025-10-13

**Authors:** Anastasiya Vasileuskaya, Agnieszka K. Szczepankowska, Jan Gawor, Magdalena Chmielewska-Jeznach, Tamara Aleksandrzak-Piekarczyk

**Affiliations:** 1 Institute of Biochemistry and Biophysics, Polish Academy of Sciences 90863, Warsaw, Poland; 2 Department of Medical Biology, Medical University of Warsaw 37803, Warsaw, Poland; Fluxus Inc., Sunnyvale, California, USA

**Keywords:** *Lactococcus lactis*, draft genome, probiotics

## Abstract

We report the draft genome sequence of *Lactococcus lactis* IBB2, a candidate probiotic strain with potential probiotic properties. The genome was sequenced using Illumina MiSeq, yielding 124 contigs totaling 2,646,375 bp. This genome provides a valuable resource for comparative genomics and probiogenomic analyses of this strain.

## ANNOUNCEMENT

*Lactococcus lactis* IBB2, a bovine milk isolate, was identified in a screening effort focused on functional probiotic traits, including potential health-related activities. To facilitate genomic mining of this strain, we sequenced its genome.

The strain was originally isolated in the late 1990s from raw bovine milk that had been aseptically collected in Poland for routine dairy microbiology studies. The sampling did not involve live animals or experimental procedures. According to ASM animal-use guidelines (https://journals.asm.org/animal-use), the isolation of *L. lactis* IBB2 from raw bovine milk is exempt from Institutional Animal Care and Use Committee review. Raw milk was transported on ice to the laboratory and processed within 6 h of collection. Ten-fold serial dilutions were prepared in sterile phosphate-buffered saline, and 100 µL aliquots of appropriate dilutions were spread on glucose-M17 agar (Oxoid). Plates were incubated aerobically at 30°C for 24–48 h. A single typical *Lactococcus* colony was picked for further purification and preservation at –80°C in GM17 broth supplemented with 20% (vol/vol) glycerol. The culture used for DNA extraction was derived from this stock after no more than two passages. Bacterial cultures were grown aerobically in glucose-M17 broth (Oxoid) for 16 hours at 30°C under static conditions. Genomic DNA was extracted from 5 mL of culture using a modified cetyltrimethylammonium bromide (CTAB)/lysozyme protocol (1), optimized to preserve high-molecular-weight DNA without commercial kits. Cells were treated with lysozyme (20  mg/mL, 37°C, 30 min), followed by proteinase K digestion and CTAB-based lysis. DNA was extracted with phenol:chloroform:isoamyl alcohol, precipitated, ethanol-washed, and treated with RNase A. DNA quality was verified by spectrofluorometry and gel electrophoresis.

Illumina sequencing libraries were prepared using the NEBNext Ultra II FS kit (New England Biolabs, UK) and sequenced on the Illumina MiSeq platform (2 × 300  bp paired-end, v3 600-cycle chemistry). Raw reads were quality-checked using FastQC v0.12.0 (2) and trimmed with fastp v0.23.2 (3). The sequencing run for IBB2 yielded 779,179 reads totaling 265,923,938 nt. The draft genome was assembled *de novo* using Unicycler v0.4.8 (https://github.com/rrwick/Unicycler), resulting in 124 contigs totaling 2,646,375  bp, with a minimum contig size of 207  bp and a maximum of 388,420  bp. The average contig length was 21,342 bp, and the GC content was 35.0%. The mean genome sequencing coverage was 98.8×. No ambiguous bases (non-ACGT) were present. N50 and L50 values were 116,610 bp and six contigs, respectively. Annotation was performed with the NCBI Prokaryotic Genome Annotation Pipeline (PGAP) v6.6 (4). PGAP annotation predicted 2,788 genes in total, including 2,650 protein-coding genes, 4 rRNA genes, 54 tRNA genes, and 3 other noncoding RNAs (Table 1). Default parameters were used for all software (FastQC v0.12.0, fastp v0.23.2, Unicycler v0.4.8, and PGAP v6.6) unless otherwise specified.

**TABLE 1:** Genome assembly and annotation statistics for *L. lactis* IBB2

Feature	Value
Total reads	779,179
Total bases	265,923,938 nt
Coverage (mean)	98.8×
Number of contigs	124
Total genome size	2,646,375 bp
Minimum contig length	207 bp
Maximum contig length	388,420 bp
Average contig length	21,342 bp
N50	116,610 bp
L50	6 contigs
GC content	35.0%
Ambiguous bases (Ns)	0
Genes (total)	2,788
Protein-coding genes (CDS)	2,650
rRNA genes (5S, 16S, 23S)	4 (1, 1, 2)
tRNA genes	54
Other noncoding RNAs	3
Pseudogenes	77

The availability of the *L. lactis* IBB2 genome will facilitate comparative genomics and probiogenomic analyses aimed at elucidating potential mechanisms underlying the probiotic properties of this strain.

## Data Availability

The draft genome sequence of *Lactococcus lactis* IBB2 has been deposited in GenBank under the accession number JBPZNJ000000000.1. Raw sequencing reads are available in the NCBI Sequence Read Archive (SRA) under the accession number SRR34752327. The associated BioProject and BioSample accession numbers are PRJNA1297673 and SAMN50232382, respectively. The assembly accession number is GCA_051904295.1.
